# p16 Stimulates CDC42-Dependent Migration of Hepatocellular Carcinoma Cells

**DOI:** 10.1371/journal.pone.0069389

**Published:** 2013-07-24

**Authors:** Ya-Wen Chen, Hsiao-Chien Chu, Ze-Shiang Lin, Wei-Jyh Shiah, Chen-Pin Chou, David S. Klimstra, Brian C. Lewis

**Affiliations:** 1 National Institute of Cancer Research, National Health Research Institutes, Miaoli, Taiwan; 2 Graduate Institute of Basic Medical Science, China Medical University, Taichung, Taiwan; 3 Program in Gene Function and Expression, University of Massachusetts Medical School, Worcester, Massachusetts, United States of America; 4 Department of Radiology, Kaohsiung Veterans General Hospital, Kaohsiung, Taiwan; 5 Department of Pathology, Memorial Sloan-Kettering Cancer Center, New York, New York, United States of America; 6 Program in Molecular Medicine, University of Massachusetts Medical School, Worcester, Massachusetts, United States of America; 7 Department of Cancer Biology, University of Massachusetts Medical School, Worcester, Massachusetts, United States of America; 8 Cancer Center, University of Massachusetts Medical School, Worcester, Massachusetts, United States of America; Johnson & Johnson Medical, China

## Abstract

Hepatocellular carcinoma (HCC) is a leading cause of cancer-related deaths worldwide. Tumor dissemination to the extra-hepatic region of the portal vein, lymph nodes, lungs or bones contributes to the high mortality seen in HCC; yet, the molecular mechanisms responsible for HCC metastasis remain unclear. Prior studies have suggested a potential link between accumulated cytoplasm-localized p16 and tumor progression. Here we report that p16 enhances metastasis-associated phenotypes in HCC cells – ectopic p16 expression increased cell migration in vitro, and lung colonization after intravenous injection, whereas knockdown of endogenous p16 reduced cell migration. Interestingly, analysis of p16 mutants indicated that the Cdk4 interaction domain is required for stimulation of HCC cell migration; however, knockdown of Cdk4 and Cdk6 showed that these proteins are dispensable for this phenomenon. Intriguingly, we found that in p16-positive HCC samples, p16 protein is predominantly localized in the cytoplasm. In addition, we identified a potential role for nuclear-cytoplasmic shuttling in p16-stimulated migration, consistent with the predominantly cytoplasmic localization of p16 in IHC-positive HCC samples. Finally, we determined that p16-stimulated cell migration requires the Cdc42 GTPase. Our results demonstrate for the first time a pro-migratory role for p16, and suggest a potential mechanism for the observed association between cytoplasmic p16 and tumor progression in diverse tumor types.

## Introduction

An estimated 750,000 new cases of hepatocellular carcinoma (HCC) are diagnosed each year, with a survival rate of less than 5%, and an average survival of less than one year after diagnosis [1]. HCC is usually associated with chronic infection by the hepatitis B virus (HBV) or hepatitis C virus (HCV), environmental carcinogens and alcohol consumption [2,3]. Among the common genetic and epigenetic alterations found in HCC are inactivating *TP53* mutations, and inactivation of the *INK4A/ARF* locus by deletion or promoter methylation [4–6]. These findings suggest important roles for the p53, p16^Ink4a^, and p14^Arf^ tumor suppressors in HCC pathogenesis.

We have previously described a HCC mouse model induced by the somatic and sporadic activation of oncogenes specifically in the liver [7]. Our data demonstrated that liver-specific *Trp53* deletion induced the development of lung metastases, the formation of which could be enhanced by concomitant deletion of *Ink4a/Arf* [8]. Furthermore, we showed that mouse HCC cell lines lacking both *Trp53* and *Ink4a/Arf* displayed increased migration and invasion abilities when compared to a mouse HCC cell line with *Trp53* deletion alone, suggesting that the *Ink4a/Arf* locus may play a role in the control of these processes [8].

The *Ink4a/Arf* locus encodes two distinct tumor suppressors – the cyclin dependent kinase (Cdk) inhibitor p16, and a protein translated from an alternative reading frame, Arf (p14 in human and p19 in mouse) – that are involved in the Rb and p53 pathways, respectively [9–11]. In agreement, mice with specific deletion of either *Ink4a* or *Arf* are tumor prone, but neither is as severely affected as animals lacking *Ink4a/Arf*, indicating that *Ink4a* and *Arf* play critical and non-redundant roles in suppressing malignancy [12]. We have previously shown that p19 regulates HCC cell invasion [13], yet whether p16 plays a similar role remained untested.

In approximately a third of human cancers, p16 is inactivated by chromosomal losses, point mutation, and/or promoter methylation [12,14]. Loss of p16 expression occurs frequently in the most common human cancers and has been associated with a poor prognosis [12]. Conversely, a growing body of data suggests that up-regulation of p16 correlates with a more aggressive phenotype in some types of tumors [15–19]. For example, over-expression and aberrant cytoplasmic localization of p16 in breast cancer is associated with accelerated tumor proliferation and a more malignant phenotype [15,16]. Therefore, elucidating whether p16 performs divergent functions during tumor initiation and tumor progression is of great importance.

In this manuscript, we show that ectopic p16 expression unexpectedly enhances HCC cell migration in transwell assays and lung colonization after tail vein injection, while RNA interference (RNAi)-mediated knockdown of p16 inhibits cell migration. We further show that p16-enhanced cell migration is dependent on its Cdk binding domain, and requires Cdc42. Intriguingly, our data also suggest a potential role for nuclear-cytoplasmic shuttling of p16 in this phenomenon. Collectively, these data suggest a novel role for p16 in stimulating the migration activity of hepatocellular carcinoma cells.

## Materials and Methods

### Cell lines

The MM189, BL322 and BL185 HCC cell lines have been previous described [8,13]. HepG2 cells, purchased from American Type Culture Collection, were cultured in Dulbecco’s Modified Eagle Medium (DMEM, Invitrogen) supplemented with 10% fetal bovine serum (FBS, Biological Industries) and antibiotics (Invitrogen).

### Ethics Statement

All animal studies were performed in strict accordance with the recommendations in the guidelines for the care and use of Laboratory Animals of National Health Research Institutes, Taiwan. The Institutional Animal Care and Use Committee (IACUC) of National Health Research Institutes approved the protocols (Protocol No:NHRI-IACUC-098055-A and NHRI-IACUC-099102-A). Animals were housed with abundant food and water. All efforts were made to minimize suffering.

### Plasmids

All cDNA expression constructs were generated in either pBabe-puro or pBabe-neo expression vectors (Addgene). cDNA encoding wild type mouse p16 was generated by reverse transcription and PCR amplification of RNA isolated from BL185 HCC cells using the Superscript III first strand synthesis system (Invitrogen) according to the manufacturer’s protocol. cDNAs encoding p16 mutants were generated by site-directed mutagenesis using PCR with mismatched annealing. HIV rev NES or SV40 NLS tagged p16 constructs were generated by PCR amplification using primers containing the NES or NLS sequences. All primer sequences are listed in Table S1. Expression constructs were transfected into the packaging cell line 293G/P, in company with Pol/GAG and pVSV-G plasmids (Clontech) using the Polyjet transfection reagent (SignaGen lab). After 48 hr incubation, viral supernatants were transferred on to target cells, and infected cells cultured in the presence of either 8 µg/ml puromycin (Calbiochem) or 0.5 mg/ml neomycin (G418, Biochrom AG). RNAi-mediated depletion was achieved by infecting cells with pLKO-based lentiviruses encoding short hairpin RNA (shRNA) targeting the mRNA (National RNAi Core Facility, Academia Sinica, Taiwan). Clones used are listed in the supplemental materials. RNAi-mediated depletion of mouse p19 was conducted by infecting target cells with MLP retroviral vectors encoding a short hairpin RNA (shRNA) targeting Arf-specific sequences (a gift from Dr. Scott W. Lowe, Cold Spring Harbor Laboratory).

### IP-Western blot analysis

Cell lysate was mixed with RIPA buffer lacking SDS and deoxycholate in company with primary anti-p16 antibody (Santa Cruz) and pre-washed Protein A/G beads (Calbiochem). Following several washes, beads were denatured in sample buffer and supernatant was saved for immunoblot analysis.

### Immunoblotting

Immunoblotting was performed as previously described [13]. Primary antibodies used are listed File S1.

### Analysis of GTPase activity

Endogenous small GTPase activity was determined using glutathione S-transferase (GST) fusion proteins containing either the p21-binding domain (PBD) of human p21-activated protein kinase 1 (Pak1) (GST-PAK1) that interacts with active forms of Cdc42 and Rac1, or the Rhotekin-binding domain (RBD) that interacts with active Rho (GST-PAK1 and GST-Rhotekin, are gifts from Dr. Lu-Hai Wang, National Health Research Institutes, Taiwan) [20,21]. Briefly, cell lysate in RIPA-buffer was incubated with pre-washed glutathione beads (Sigma) bound with either GST-PAK1 or GST-Rhotekin. After washing, boiling in sample buffer eluted bound proteins, and the supernatant loaded on to SDS-PAGE. Immunoblotting was performed with anti-Rho (05-778, Millipore), anti-Cdc42 (07-1466, Millipore) and anti-Rac1 (05-389, Millipore) antibodies. GTPγS (Millipore) served as a positive control to maintain the active forms of GTPases in the reaction. GST detection by anti-GST antibody (sc-459, Santa Cruz) served as a loading control. The protein intensity was measured and quantified using ImageJ software.

### Cell proliferation

Cell proliferation analysis was performed as previously described [8]. Experiments were repeated at least three times.

### Soft agar assay

Soft agar assays were performed as previously described [22]. The number of colonies larger than 25 µm in diameter present within 20 microscopic fields was counted under a light microscope. All experiments were performed in triplicate and repeated a minimum of three times.

### In vitro migration and invasion assays

Migration and invasion assays were performed as described in our previous studies [13]. The number of migrated cells was determined by counting the cell numbers in 5 fields/per insert at 100X magnification. The mean number of migrated cells was determined by averaging the number of migrated cells in two independent inserts. The relative migration activity was determined by normalizing the mean number of migrated cells in experimental cells to that of vector controls, with the migration activity of the control cells set to 1. Invasion assays were performed using transwells with matrigel coating (Becton Dickinson). The number of invaded cells was determined by counting the cell numbers in 5 fields/per insert at 100X magnification. The mean number of invaded cells was determined by averaging the number of invaded cells in two independent inserts. The relative invasion activity was determined by normalizing the mean number of invaded cells in experimental cells to that of vector controls, with the invasion activity of the control cells set to 1. Each experiment was repeated at least three times. Data shown are from representative experiments.

### Immunofluorescence (IF)

IF was performed as described previously [13]. Primary antibody used: anti-p16 (sc-1207, Santa Cruz).

### Lung colonization assay

Lung colonization assay was conducted as described previously [8]. After 25-28 days, the lungs were harvested for paraffin embedding, sectioning, and histological examination after H&E staining. 4-5 animals were included in each group. The tumor area was calculated using ImageJ software.

### Immunohistochemical staining of human hepatocellular carcinomas

Tissue microarrays (TMAs) of resected cases of human hepatocellular carcinomas were previously described [23]. Briefly, triplicate 0.6 mm cores of carcinomas and matched non-neoplastic liver samples from 124 hepatocellular carcinomas (18 fibrolamellar carcinomas and 106 conventional hepatocellular carcinomas) were punched and embedded in donor blocks. Immunohistochemical staining for p16 was performed using a monoclonal p16 antibody, clone INK4A (MTM Laboratories, Heidelberg, Germany), diluted 1:5, with standard techniques on the Ventana Discovery XT (Ventana Corporation, Tucson AZ) automated immunostainer. Samples of gastric and endometrial adenocarcinomas known to overexpress p16 were used as positive controls. The stains were interpreted as positive if there was distinct cytoplasmic or nuclear reactivity in the neoplastic cells, greater than any background staining in the matched non-neoplastic liver, in more than 20% of the cells within the different cores of tumor. Cytoplasmic and nuclear staining were separately recorded. Survival information for the patients was obtained from the clinical records.

### Statistical analysis

Statistical calculation was performed using student t test (Graphpad software, San Diego, CA). Findings with a *p*-value of less than 0.05 were considered statistically significant. *, *p*<0.05; **, *p*<0.01; ***, *p*<0.001.

## Results

### p16 expression enhances HCC cell migration

**Figure 1:**
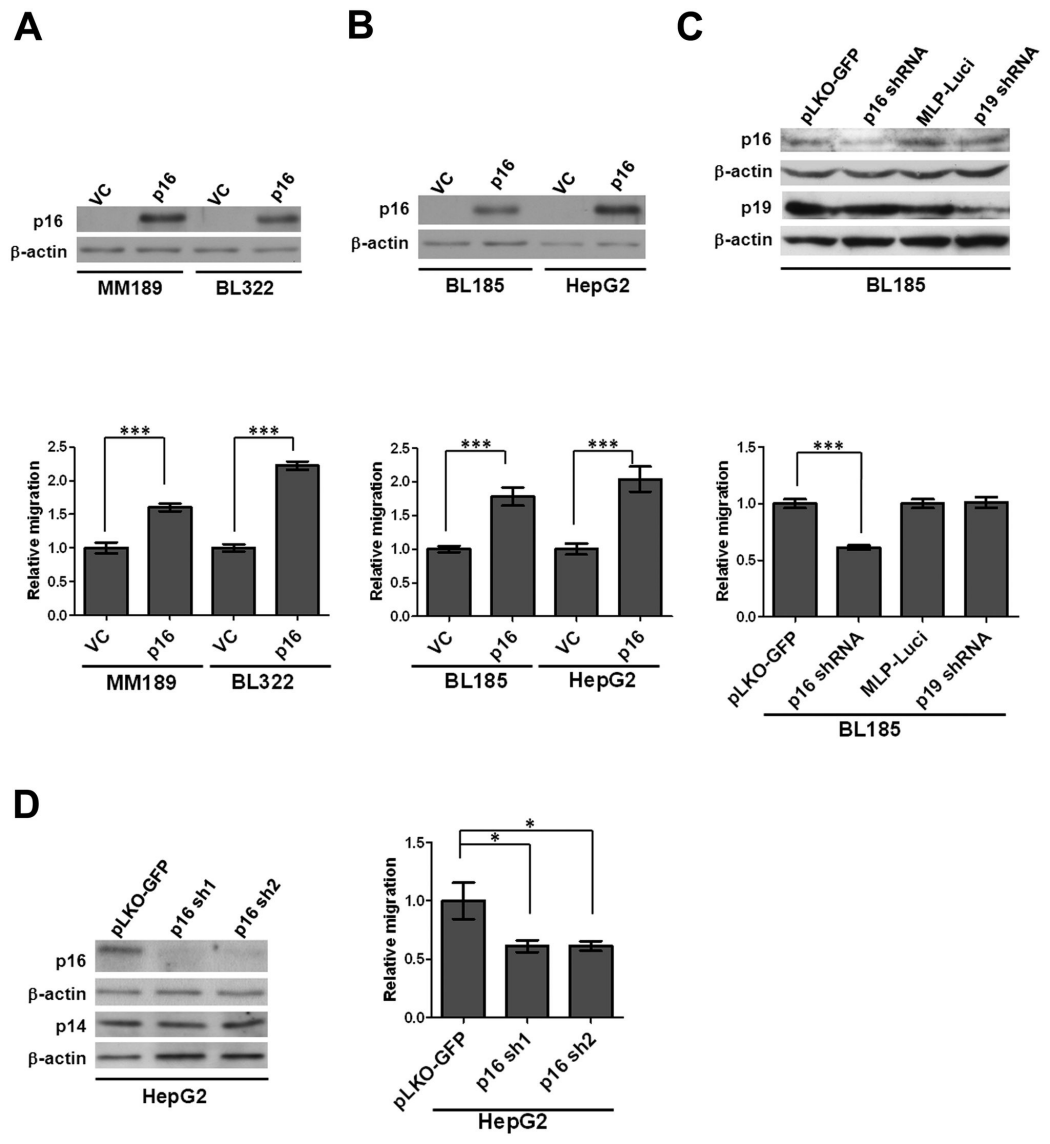
p16 enhances HCC cell migration. (**A**) Upper panel: Immunoblot confirming ectopic p16 expression in MM189 and BL322 mouse HCC cells infected with either pBabe-puro retrovirus (Vector control, VC) or pBabe-puro retrovirus encoding wild type p16 (p16). β-actin serves as a loading control. Lower panel: Migration activity of MM189 and BL322 cells expressing p16, and their vector controls. Data are from a representative experiment performed in duplicate. Bar, SEM. (**B**) Upper panel: Immunoblot detection of ectopic p16 protein in BL185 and HepG2 HCC cells. Lower panel: Migration activity of BL185 and HepG2 cells expressing ectopic p16, and their vector controls. Data are from a representative experiment performed in duplicate. Bar, SEM. (**C**) Upper panel: Immunoblot detection of specific knockdown of p16 and p19 in BL185 HCC cells. pLKO-GFP is the non-silencing control for the p16-targeting shRNA. MLP-Luci is the non-silencing control for the p19-specific shRNA. Lower panel: Migration activity of BL185 cells with p16- or p19-specific knockdown relative to their non-silencing controls. Data are from a representative experiment performed in duplicate. Bar, SEM. (**D**) Left panel: Immunoblot detection of p16-specific knockdown in HepG2 cells infected with lentiviral vectors encoding p16-targeting shRNA, or a non-silencing control pLKO-GFP. Right panel: Migration activity of HepG2 cells with p16 knockdown relative to cells expressing the non-silencing control (pLKO-GFP). Data are from a representative experiment performed in duplicate. Bar, SEM. *, *p*<0.05; **, *p*<0.01; ***, *p*<0.001.

Our previous studies suggested that the *Ink4a/Arf* locus plays a key role in regulating HCC cell migration, invasion, and metastasis [8,13]. Therefore, we ascertained whether loss of p16 was required for cellular phenotypes associated with tumor metastasis [15,16,24–26]. The murine HCC cell lines MM189 and BL322, which are deleted at both *Trp53* and *Ink4a/Arf*, were infected with a retroviral vector encoding mouse p16, or empty vector as a control, and ectopic p16 expression confirmed by immunoblot (Figure 1A). We observed that MM189 and BL322 cells with ectopic p16 expression displayed increased migration in a transwell assay when compared with their corresponding controls (Figure 1A). To exclude potential retroviral mutagenesis effects, the experiments were repeated with at least two independent batches of infected cells and similar results obtained. We additionally tested whether ectopic expression of p16 similarly enhanced cell invasion. However, while ectopic p16 enhanced cell migration in both low and high passage MM189 cells, it induced cell invasion in high passage cells, but not low passage cells (Figure S1A). We therefore focused our studies on the characterization p16’s involvement in HCC cell migration.

To confirm that p16-enhanced migration occurs in additional mammalian cells, including human cells, the human hepatoblastoma cell line HepG2 and the BL185 murine HCC cell line that retain functional *Ink4a/Arf*, were infected with a retroviral vector encoding mouse p16, or an empty vector control, and ectopic p16 expression confirmed by immunoblot (Figure 1B). Ectopic p16 increased cell migration in both HepG2 and BL185 cells when compared with their corresponding controls (Figure 1B), suggesting that p16 enhanced cell migration is not cell line- or species-specific.

To determine whether loss of p16 reduces HCC cell migration, we infected the BL185 HCC cell line with viral vectors encoding shRNAs against mouse p16 or p19 and specific knockdown confirmed by immunoblot (Figure 1C). Reduced p16 levels resulted in a dramatic decrease in migration activity; however, consistent with our published data [13], this reduction was not observed in BL185 cells with p19 knockdown (Figure 1C). Likewise, RNAi-mediated depletion of p16 in HepG2 cells (Figure 1D) reduced migration activity in that cell line (Figure 1D). Taken together, our data demonstrate that expression of p16 is necessary and sufficient for enhanced HCC cell migration.

Analysis of pRb expression demonstrated that MM189 cells do not express pRb, whereas HepG2 cells are pRb-positive (Figure S1B). These data suggest that p16 induces cell migration independent of pRb status. Consistent with this, knockdown of pRb in HepG2 p16 cells (Figure S1C) did not impact cell migration (Figure S1D).

### p16 enhances the ability of HCC cells to colonize the lungs

**Figure 2:**
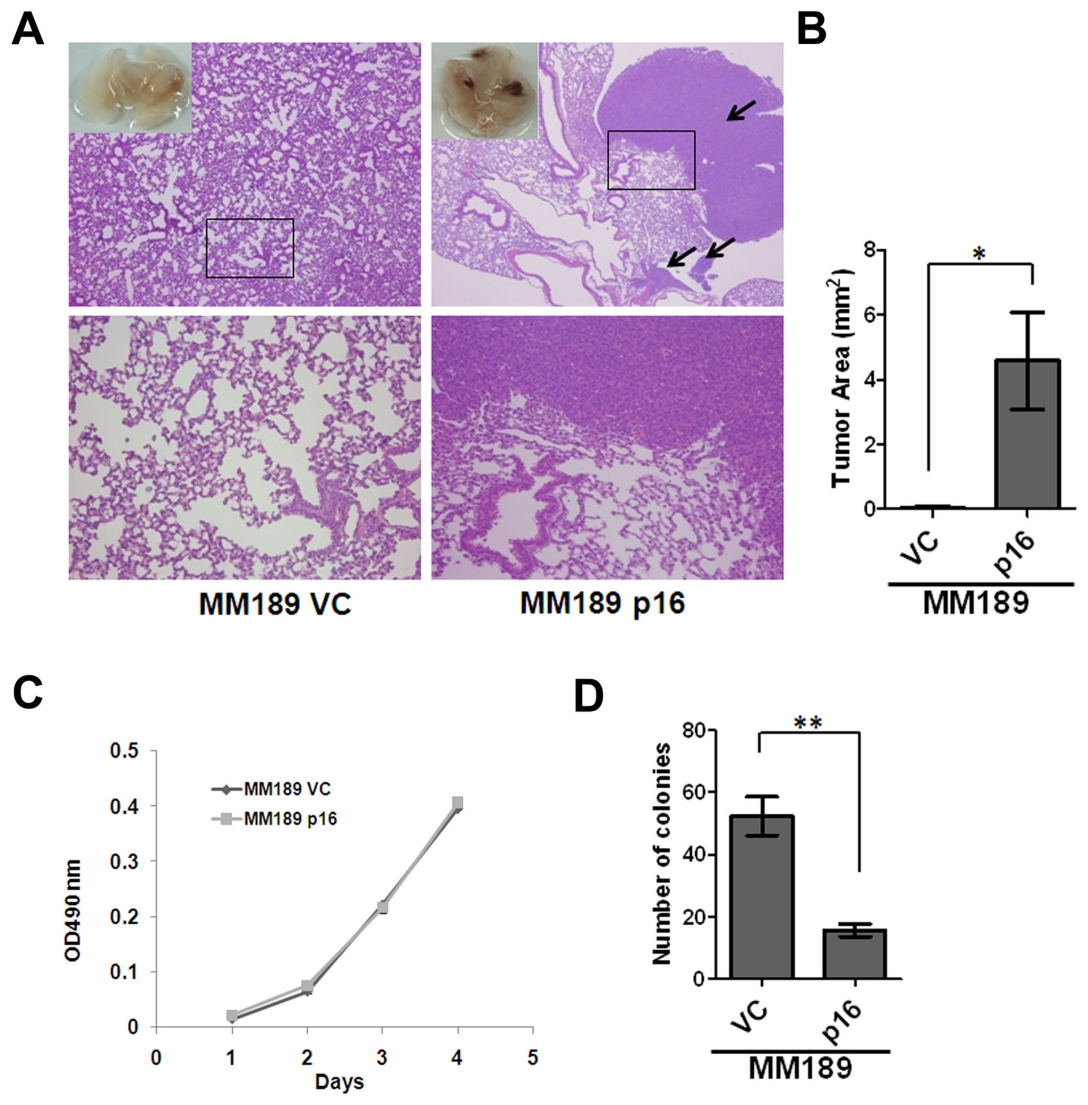
p16 enhances lung colonization by HCC cells. (**A**) Representative lung fields of nude mice after the delivery, via tail vein injection, of MM189 cells with ectopic p16 expression (MM189 p16) or vector controls (MM189 VC). The boxed area in the upper panel is shown at higher magnification in the lower panel. (**B**) Quantification of the total area of the lung lesions in mice injected with MM189 p16 or MM189 VC cells. Bar, SEM. (**C**) Representative cell proliferation assay for MM189 p16 and MM189 VC cells. (**D**) Representative anchorage-independent growth assay for MM189 VC and MM189 p16 cells. Bar, SEM. *, *p*<0.05; **, *p*<0.01; ***, *p*<0.001.

To ascertain whether the effect of p16 on HCC cell migration correlated with an *in vivo* phenotype, we determined the ability of MM189 cells with ectopic p16 expression, or empty vector controls, to colonize the lungs after tail vein injection. We observed that p16 re-expression led to increased colony formation in the lungs (100% versus 25% for vector controls) and a dramatic increase in tumor area (Figure 2A and 2B). p16-expressing MM189 cells formed lung lesions with an average area of 4.583 ± 1.505 mm^2^ compared with 0.0491 ± 0.0491 mm^2^ for controls (Figure 2B). Thus, p16 enhances the ability of HCC cells to colonize the lungs *in vivo*.

We further asked whether p16 plays critical roles in other tumor-associated phenotypes in HCC cells. Measurement of cell proliferation in MM189 p16 versus MM189 vector control cells (Figure 2C), and HepG2 p16 versus HepG2 control cells (Figure S1E), demonstrated that p16 inhibits cell proliferation in a pRb-dependent manner. Next, we determined whether p16 affected the ability of MM189 cells to undergo anchorage-independent growth and found that p16 expression reduces colony formation in soft agar (Figure 2D). Consistent with this observation, RNAi-mediated knockdown of p16 in HepG2 cells enhances soft agar colony formation (Figure S1F). Thus, while p16 enhances HCC cell migration, it retains other classical tumor suppressor functions in these cells. These data suggest that p16 may regulate cell migration and cell cycle progression through separate mechanisms.

### Cdk4 and Cdk6 are dispensable for p16-enhanced migration

**Figure 3:**
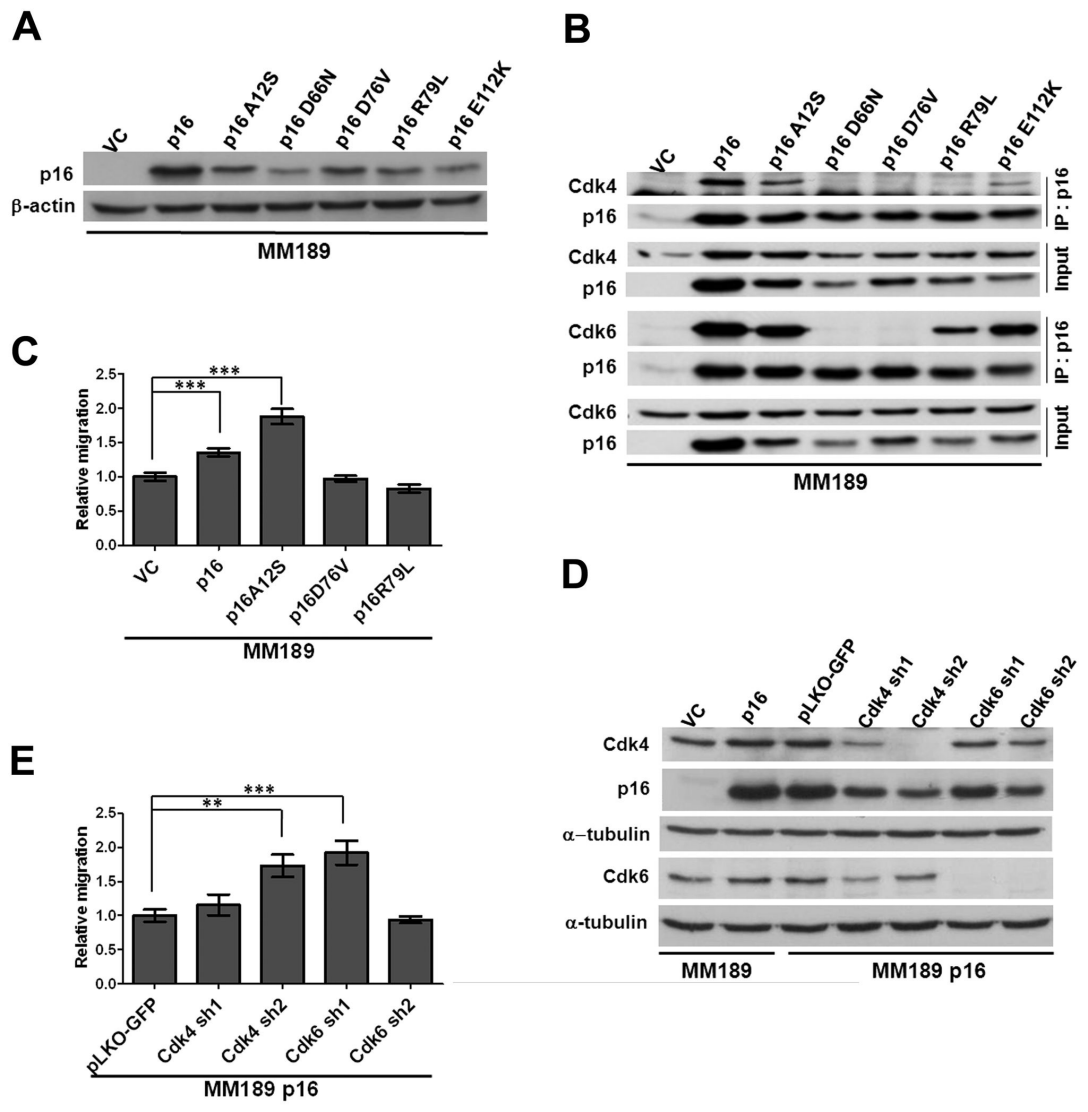
Cdk4 and Cdk6 are dispensable for p16-enhanced migration. (**A**) Immunoblot detection of p16 mutants in MM189 cells. (**B**) IP-Western Blot detection of binding of p16 mutants to Cdk4 and Cdk6. (**C**) Migration activity of MM189 cells expressing listed p16 mutants. Bar, SEM. (**D**) Immunoblot for Cdk4 or Cdk6 knockdown in MM189 p16 cells. (**E**) Migration activity of MM189 p16 cells with Cdk4 or Cdk6 knockdown relative to that of cells infected with a non-silencing control, pLKO-GFP. Bar, SEM. *, *p*<0.05; **, *p*<0.01; ***, *p*<0.001.

p16 binds to Cdk4 and Cdk6 to inhibit phosphorylation of pRb and regulate the progression of the cell cycle. To investigate whether interaction with Cdk4 or Cdk6 is involved in p16-enhanced migration, we generated several p16 mutants, some originally identified in human tumors, introduced them into MM189 cells by retroviral transduction, and confirmed expression by immunoblotting with p16-specific antibodies (Figure 3A). We next assessed the ability of the mutants to bind to Cdk4 and Cdk6 in co-immunoprecipitation experiments, and observed three classes of mutants: the D66N and D76V mutants that fail to interact with both Cdk4 and Cdk6; the A12S and E112K mutants that retain interaction with both Cdks; and the R79L mutant that weakly binds Cdk6 but fails to interact with Cdk4 (Figure 3B). We next tested representative mutants for their effect on HCC cell migration. While the A12S mutant induced migration as effectively as wild type p16 in MM189 cells, the D76V and R79L mutants failed to do so (Figure 3C). Thus the Cdk4 binding region of p16 is required for its ability to induce the migration of HCC cells.

To directly test the requirement for Cdk4 and Cdk6 in p16-induced cell migration, we knocked down Cdk4 and Cdk6 expression in MM189 cells and confirmed knockdown by immunoblot (Figure 3D). Reduced Cdk4 or Cdk6 levels did not impair p16-mediated migration, suggesting that Cdk4 and Cdk6 are dispensable for p16-enhanced migration even though the Cdk interaction domain is required (Figure 3E).

### p16 is localized in the cytoplasm in human HCC

**Figure 4:**
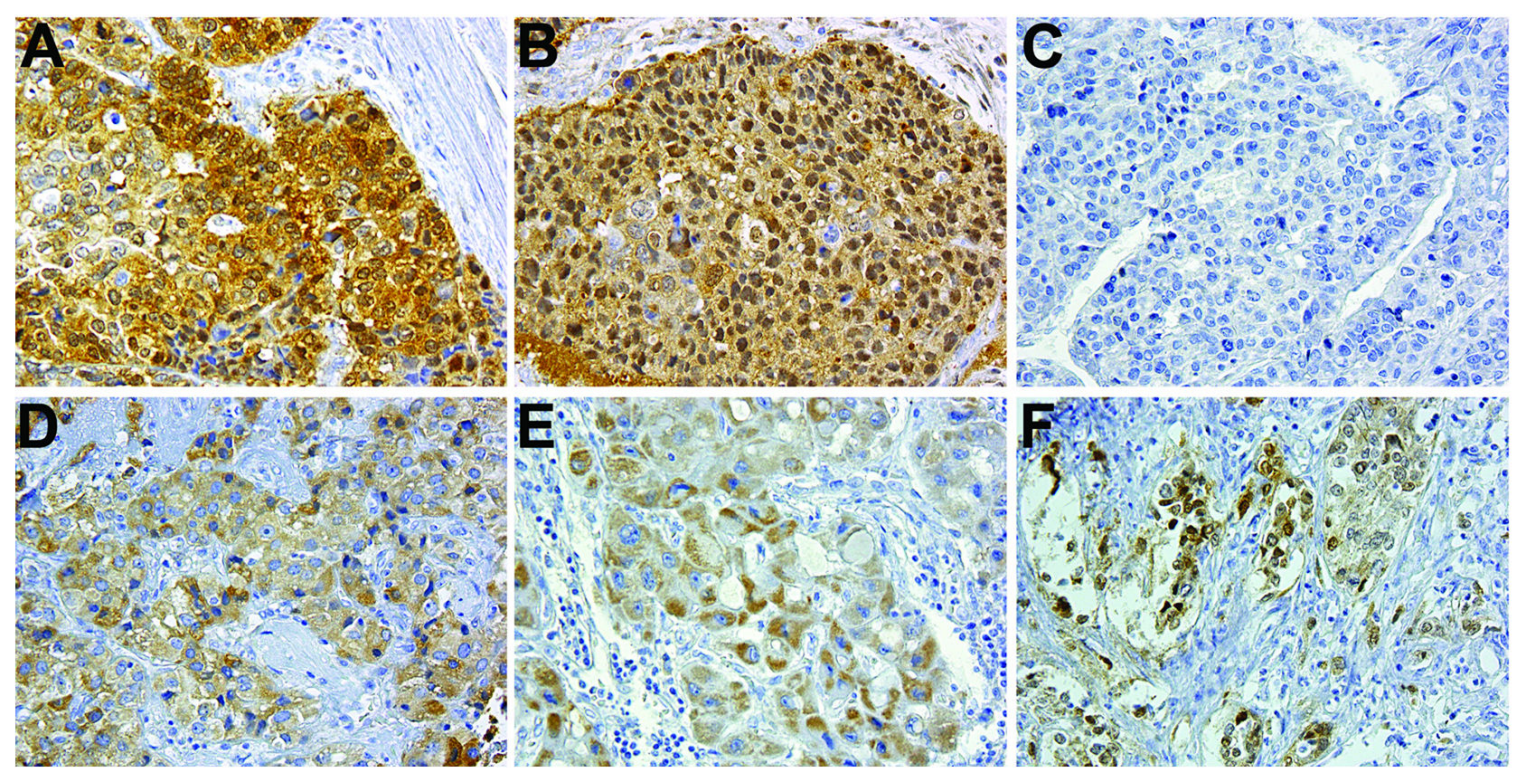
Human HCC samples display cytoplasmic localization of p16. Control gastric (**A**) and endometrial (**B**) carcinoma samples displaying nuclear and cytoplasmic staining for p16. (**C**–**F**) Representative images of HCC samples displaying different levels and localization of p16 staining. Note the absence of nuclear staining in panels (**D** and **E**). All images are at 200X magnification.

Prior studies suggested that accumulated cytoplasm-localized p16 could be identified in certain tumor types and is associated with tumor progression and poor prognosis [15,16]. Moreover, a survey of prior gene expression profiling studies in Oncomine suggested a correlation between elevated *CDKN2A* mRNA levels and increased tumor grade [24–27]. Therefore, to determine whether aberrant cytoplasmic localization of p16 also occurs in hepatocellular carcinoma, we stained a tissue microarray (TMA) containing human liver tumor samples. 124 tissue cores on the array could be evaluated. Of these, 18 were fibrolamellar carcinomas, and 3 of these stained positive for p16. Of the 106 HCC cases on the TMA, 90 did not display p16 staining above background (Figure 4C), whereas 16 cases displayed detectable p16 staining (Figure 4D–F). In most cases (12), the p16 staining was evident in 21-80% of the cancer cells, whereas 4 samples displayed focal staining in less than 20% of cells. Interestingly, of the p16-positive HCCs, 15 displayed predominantly cytoplasmic staining (Figure 4D, E), while only a single tumor displayed strong nuclear staining (Figure 4F). Importantly, gastric (Figure 4A) and endometrial (Figure 4B) carcinoma samples on the TMA displayed either nuclear p16 staining or both nuclear and cytoplasmic staining, suggesting that the cytoplasmic staining pattern observed in the HCC samples is not artifactual. Immunohistochemical staining of paired HCC and normal tissues demonstrated cytoplasmic staining of p16 in the tumor but nuclear staining in normal hepatocytes (arrows, Figure S2). Thus, p16 is predominantly localized to the cytoplasm in HCC tissue.

### Nuclear-cytoplasmic shuttling of p16 is required for enhanced migration

**Figure 5:**
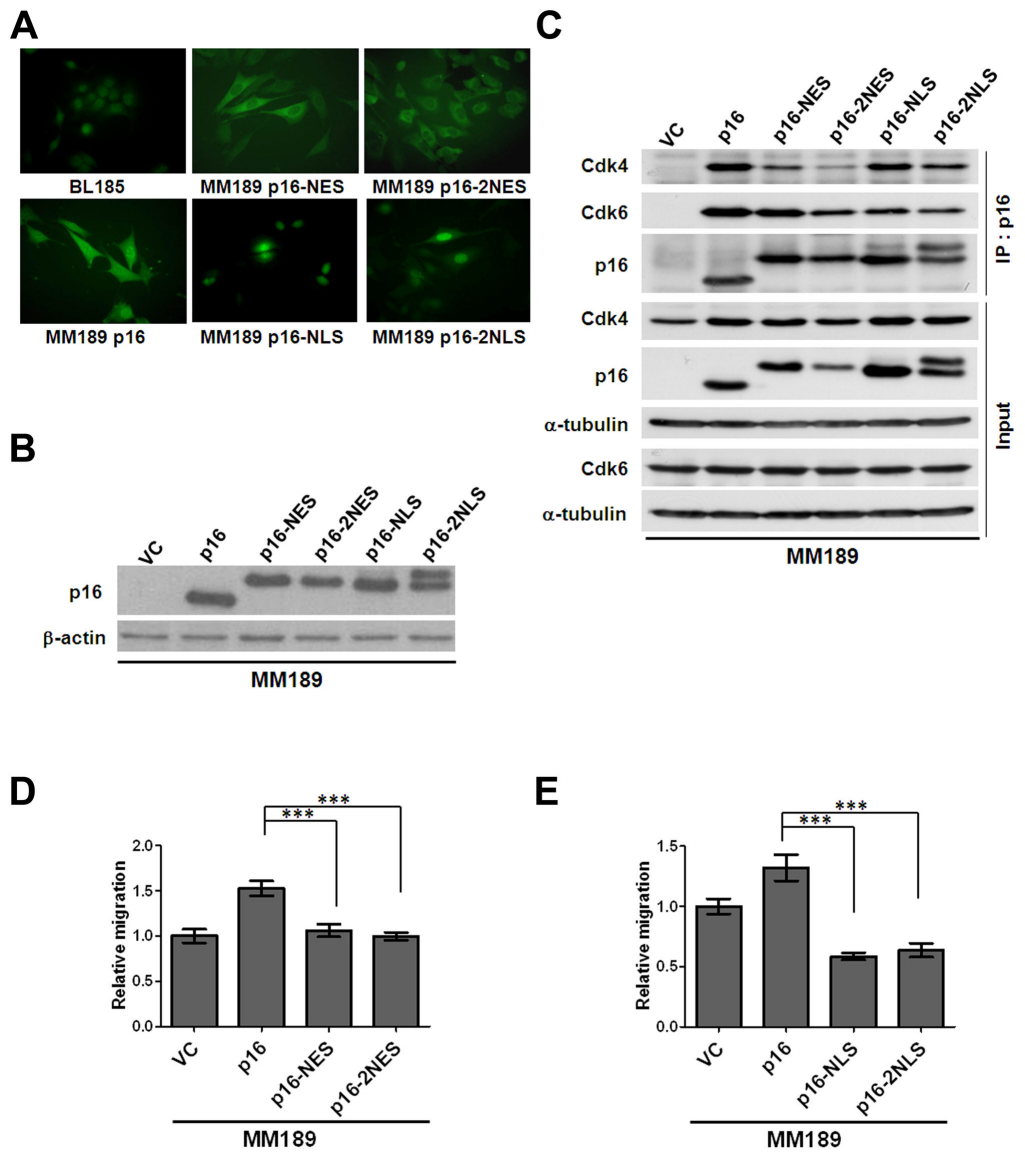
Nuclear-cytoplasmic shuttling is involved in p16-induced cell migration. (**A**) Immunofluorescent detection of sub-cellular localization of p16 proteins. p16-NES, p16 tagged with NES at the C-terminus; p16-2NES, p16 tagged with NES at both N- and C-termini; p16-NLS, p16 tagged with NLS at the C-terminus; p16-2NLS, p16 tagged with NLS at both N- and C-termini. (**B**) Immunoblot detection of p16 proteins tagged with NES or NLS sequences. β-actin serves as a loading control. (**C**) IP-Western blot confirming the ability of NES- or NLS-tagged p16 to bind to Cdk4 and Cdk6. (**D**) Migration activity of MM189 cells expressing NES-tagged p16 proteins. Bar, SEM. (**E**) Migration activity of MM189 cells expressing NLS-tagged p16 proteins. Bar, SEM. *, *p*<0.05; **, *p*<0.01; ***, *p*<0.001.

Since our immunostaining data indicated that p16 was localized predominantly within the cytoplasm in HCC tissues, we ascertained the cellular localization of p16 in the HCC cell lines used in our prior experiments. Endogenous p16, present in BL185 cells, is predominantly localized within the nucleus (Figure 5A). However, ectopically expressed p16 in this cell line is distributed in both the nucleus and cytoplasm (Figure S3A). Similarly, reintroduction of p16 into the *Ink4a/Arf* null MM189 HCC cell line resulted in localization of the ectopic protein within the nucleus and cytoplasm (Figure 5A). Interestingly, using leptomycin B mediated blockade of nuclear export, we demonstrated that the ectopic p16 protein in MM189 cells shuttles between the nucleus and cytoplasm through an active transport process (Figure S3B). These data, coupled with the identification of cytosol-localized p16 in tumor samples, raised the possibility that the cytoplasmic localization of p16 contributes to its ability to induce migration in HCC cells. However, p16 does not have a recognizable nuclear localization signal (NLS) or a nuclear export signal (NES). Therefore, to test whether sub-cellular localization of p16 contributes to cell migration, we generated expression constructs encoding p16 tagged with the HIV Rev NES at either the C-terminus or both N- and C-termini, and constructs encoding p16 tagged with the Simian Virus 40 T antigen NLS at either the C-terminus or both N- and C-termini. Immunoblotting confirmed the stability of the tagged proteins (Figure 5B) and immunofluorescence staining confirmed their restriction to the appropriate sub-cellular compartment (Figure 5A).

We additionally confirmed that the NLS- and NES-tagged p16 proteins retained the ability to interact with Cdk4 and Cdk6 in co-IP experiments (Figure 5C). In transwell migration assays, we found that cytosol-localized p16 failed to stimulate cell migration (Figure 5D). Interestingly, nuclear-restricted p16 likewise failed to induce cell migration in MM189 cells (Figure 5E). These data suggest that restriction of p16 to a specific cellular compartment impairs its ability to stimulate HCC cell migration, and further suggest that nuclear-cytoplasmic shuttling of p16 plays an important role in p16-induced cell migration in HCC cells.

### Increased Cdc42 and Rac1 GTPase activity in p16-enhanced migration

**Figure 6:**
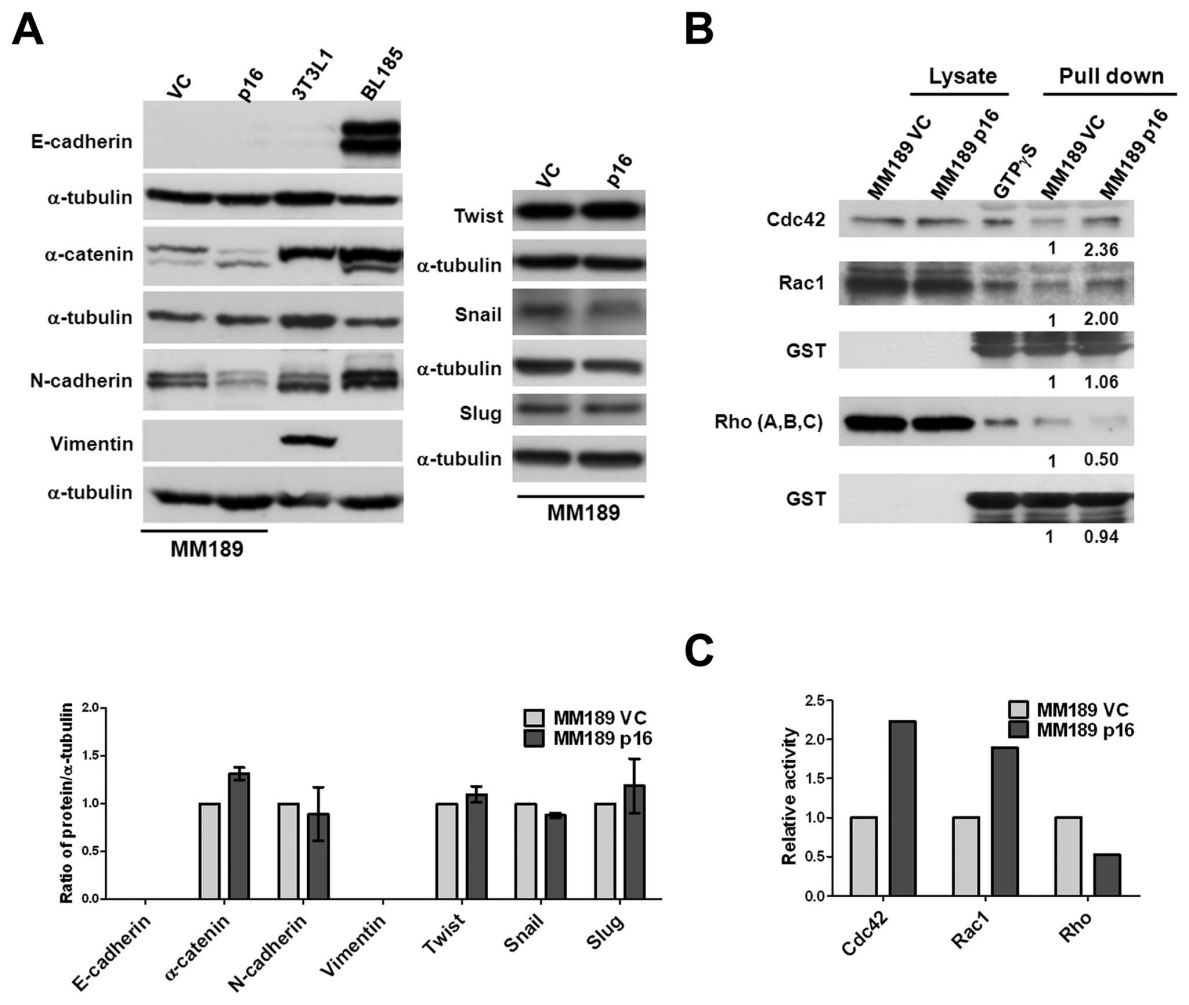
Enhanced Cdc42 and Rac1 activity in p16-expressing HCC cells. (**A**) Immunoblot analysis of epithelial and mesenchymal proteins and EMT-associated transcription factors in MM189 p16 and MM189 VC cells. BL185 cells and 3T3L1 cells serve as positive controls for the expression of E-cadherin and vimentin, respectively. α-tubulin serves as a loading control. The lower panel shows the quantification of 3 independent immunoblots. (**B**) Immunoblot detection of active Cdc42, Rac1, and Rho proteins bound by GST-Pak1 and GST-Rhotekin. GTPγS serves as a positive control to maintain the active form of GTPase in the reaction. Immunoblotting of whole cell lysate serves as an input control, and detection of GST serves as a loading control. (**C**) The relative density of the bands corresponding to Cdc42, Rac1 and Rho in MM189 p16 cells and control MM189 VC cells.

Tumor cell migration is often associated with the occurrence of an epithelial-to-mesenchymal transition (EMT), and the activation of the small GTPases Rho, Cdc42 and Rac1 [28,29]. We therefore determined whether any of these phenomena are associated with p16-induced cell migration. Immunoblotting showed that p16 expression in MM189 cells does not affect the levels of epithelial proteins such as α-catenin and E-cadherin, or mesenchymal markers, including N-cadherin and Vimentin (Figure 6A). Further, p16 re-expression did not significantly increase the level of EMT-related transcription factors, such as Twist, Snail and Slug (Figure 6A). In contrast, our recently published data demonstrated that Klf4 regulates an EMT in this cell line [30]. Thus, p16-induced migration does not involve an EMT.

Next, cell lysates were analyzed for evidence of activation of small GTPases via the ability of GST-fused Pak1-PBD to capture active Rac1 (Rac1-GTP) or Cdc42 (Cdc42-GTP) molecules and GST-fused Rhotekin-RBD to capture active Rho (Rho-GTP) molecules in a pull down assay [20,21]. As compared to the vector control, expression of p16 resulted in the robust induction of Cdc42-GTP and Rac1-GTP with 2.23- and 1.89-fold increases, respectively, but reduced Rho-GTP levels by almost 2-fold (Figure 6B and 6C), suggesting that the balance of Cdc42, Rac1 and Rho proteins, mediates p16-induced cell migration.

**Figure 7:**
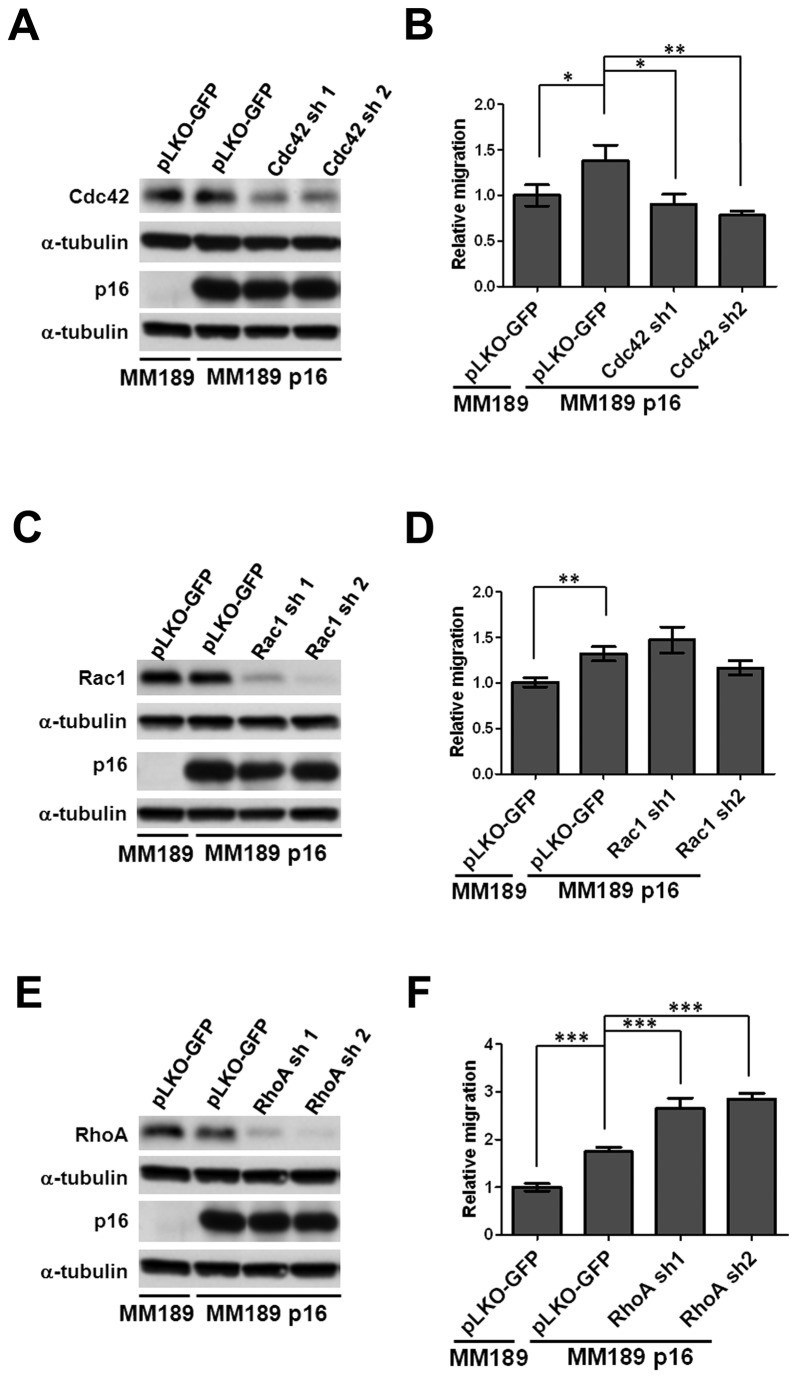
Cdc42 is required for p16-mediated migration. Immunoblot analysis for knockdown of (**A**) Cdc42, (**C**) Rac1, and (**E**) RhoA in MM189 p16 cells. Cells infected with a vector encoding an shRNA targeting GFP serve as a non-silencing control (pLKO-GFP). α-tubulin serves as a loading control. Migration activity of cells with knockdown of (**B**) Cdc42, (**D**) Rac1, and (**F**) RhoA relative to MM189 cells infected with a non-silencing control. Data are from a representative experiment performed in duplicate. Bar, SEM. *, *p*<0.05; **, *p*<0.01; ***, *p*<0.001.

We next determined whether these small GTPases are required for p16-mediated migration by targeting individual GTPases with specific shRNAs. We found that knockdown of Cdc42 strongly inhibited p16-mediated cell migration (Figure 7A, B), while depletion of Rac1 had only a negligible effect (Figure 7C, D). By contrast, knockdown of RhoA enhanced p16-mediated migration (Figure 7E, F). We observed similar effects in cells treated with the Rho inhibitor Y27532 and a Rac1 inhibitor (Figure S4A and B). These data indicate that Cdc42 is a positive mediator of p16-induced HCC cell migration, while RhoA inhibits this phenotype.

## Discussion

*INK4A* encodes p16, an inhibitor of Cyclin D/Cdk complexes. p16 acts to constrain Cdk-mediated phosphorylation of pRb, thereby regulating transition through the G_1_ phase of the cell cycle. Consistent with this function, p16 is a bona-fide tumor suppressor and loss of p16 function is a common event in human cancers [31–33]. However, emerging data have suggested that overexpression and abnormal cytoplasmic localization of p16 may correlate with tumor grade, tumor progression and/or poor prognosis in multiple tumor types including HCC [15–19,24–26,34]. These findings suggest that, in certain contexts, p16 may potentially play pro-tumorigenic roles as well; however, prior to our study, this had not been experimentally demonstrated. While the antithesis of the current view of p16 function, context-dependent pro- and anti-tumorigenic functions have been demonstrated for several proteins involved in tumorigenesis including Notch and TGF-β [35,36].

Consistent with previously published findings in other tumor types in which p16 overexpression has been observed, our immunostaining of an HCC tissue microarray demonstrated that while most HCC samples were negative for p16 staining, all of but one of the tumors with p16 immunoreactivity displayed predominantly cytoplasmic staining, whereas other tumor types on the array displayed a predominantly nuclear staining pattern. Importantly, prior studies have indicated that the presence of cytoplasmic p16 correlated with progressive disease and poor prognosis [15,16,34]. However, we did not identify a correlation between p16 levels and patient survival. While the five-year survival rate was greater in p16-negative HCC samples compared to the p16-positive samples included on our TMA (35% vs. 31%), this difference was not significant. Potentially, this result reflects the tissue samples selected for inclusion on the TMA. Our experimental data on the impact of p16 on HCC cell migration and lung colonization suggest that p16 may impact HCC metastasis; however, the samples on the TMA were all obtained from patients who underwent surgical resection, and therefore by definition did not have documented disseminated disease at the time of surgery. Future analysis of metastatic samples may yield a correlation with p16 expression.

*INK4A* is frequently inactivated by a variety of mechanisms in HCC illustrating its role as a tumor suppressor in this malignancy [5,6]. Interestingly, p16 was previously shown to inhibit the migration and invasion of breast cancer and other cells in a manner dependent on its interaction with Cdk6 [37–39]. In these prior studies, the ectopic p16 was predominantly localized to the nucleus, whereas in HCC cells ectopic p16 appears to be primarily cytoplasmic, consistent with our immunostaining data of HCC samples. Here, by contrast, we demonstrated that p16 is a positive regulator of HCC cell migration – ectopic expression of p16 enhances cell migration, whereas RNAi-mediated knockdown of p16 inhibits this phenotype. Importantly, these results are reproducible in multiple HCC and hepatoma cell lines of human and mouse origin, demonstrating the significance of these findings. Significantly, we find that increased p16 expression restricts HCC cell proliferation in a pRb-dependent manner, and that p16 inhibits other transformation-associated phenotypes, such as soft agar colony formation, consistent with its known tumor suppressor function. Thus, the effect of p16 on HCC cell migration is specific, and likely does not reflect an experimental artifact resulting from manipulation of p16 levels.

Interestingly, our data suggest that nuclear-cytoplasmic shuttling of p16 may be involved in its regulation of cell migration, as sequestration in either compartment impaired p16-induced migration. How this shuttling occurs, and how it impacts migration, remains unclear. In addition, p16-stimulated cell migration is not affected by pRb expression status. Moreover, the ability of p16 to regulate HCC cell migration does not depend on regulation of Cdk4 and Cdk6 activity, and p16 stimulates migration in cell lines in which it inhibits anchorage-independent growth. Together, these data suggest that p16 impacts cell migration via mechanisms independent of those used to regulate cell cycle progression. What are these mechanisms? Intriguingly, we found that ectopic p16 expression resulted in an increase in Cdc42 and Rac1 activation, along with a concomitant decrease in Rho activity. Furthermore, RNAi-mediated inhibition of Cdc42 reduced, while knockdown of RhoA enhanced, p16-induced cell migration. Together, these findings suggest that regulation of Rho family GTPases activity may be one mechanism.

In addition, mutation of the Cdk4-binding region of p16 demonstrated that this domain is required for its ability to stimulate cell migration, suggesting that important mediators of p16’s ability to induce cell migration also bind to this region of the protein. Identifying these potential binding proteins is critical to understanding the mechanism by which p16 stimulates cell migration, and experiments are ongoing to address this issue. Published work by Souza-Rodrigues et al has suggested that p16 interacts with various actin and tubulin proteins [40]. However, these interactions were not validated in vivo, and it remains to be determined whether these are true interactions, or background identification of abundant cellular proteins by mass spectrometry.

Of note, prior work from Roberts and colleagues identified a similar pro-migration phenotype induced by another Cdk inhibitor, p27 [41,42]. They additionally found that p27-induced migration involved its interaction with the RhoA GTPase and resulting inhibition of RhoA activation [41]. Our data suggest that a similar mechanism may be at work with regards to p16-induced cell migration as well. However, whereas a direct interaction was observed between p27 and RhoA, we have been unable to observe a direct interaction between p16 and either Cdc42 or RhoA. Therefore it remains to be elucidated whether the regulation of these GTPases downstream of p16 occurs via direct or indirect mechanisms.

Why might p16 stimulate cell migration, and in what contexts? Proper organ and tissue development requires the coordinated regulation of cell cycle exit, cellular differentiation, and cell migration. Indeed, p27 has been shown to coordinately regulate cell cycle exit and neuronal cell differentiation and migration [42]. Thus, the pro-migratory effects of p16 may reflect a previously undiscovered role in a similar process. Tumors have likely co-opted this normal developmental process – by expressing high levels of p16 in the cytosol, the cell cycle arrest function of p16 is abrogated and the pro-migratory effects of p16 are enhanced. Again, work on p27 provides supporting evidence as cytosol-sequestered p27 stimulates the migration of HepG2 cells [43,44]. However, much needs to be done to understand p16-regulated cell migration. Indeed, the ability of p16 to induce cell migration in other tumor cell types in which cytoplasmic accumulation is observed will need to be demonstrated, and the detailed mechanisms that regulate this migration elucidated. Nonetheless, the data presented herein demonstrate a novel function of p16 and highlight the complexity regarding tumor initiation and progression.

## Supporting Information

File S1 Supplementary methods.
(DOC)

Table S1 PCR primers used in vector construction.
(DOCX)

Figure S1 (**A**) Migration and invasion activity of low and high passage MM189 cells expressing p16, and their vector controls. Data are from representative experiments performed in duplicate. Bar, SEM. (**B**) Immunoblot detection of pRb expression (anti-Rb, sc-102, Santa Cruz) in MM189, BL322 and HepG2 cells. α-tubulin serves as a loading control. (**C**) Immunoblot detection of pRb knockdown in HepG2 cells with ectopic p16 expression (HepG2 p16). α-tubulin serves as a loading control. (**D**) Migration activity of HepG2 cells with ectopic p16 expression (HepG2 p16) following pRb knockdown, relative to cells expressing a non-silencing control (pLKO-GFP). Data are from a representative experiment performed in duplicate. Bar, SEM. (**E**) Representative cell proliferation assay for HepG2 with p16 ectopic expression (HepG2 p16) and vector control (HepG2 VC). Bar, SEM. (**F**) Representative soft agar assay for HepG2 cells with knockdown of p16, as well as the non-silencing control. Bar, SEM. *, *p*<0.05; **, *p*<0.01; ***, *p*<0.001.
(TIF)

Figure S2 Immunohistochemical staining of paired HCC tissue and normal liver demonstrates cytoplasmic localization of p16 in the HCC specimen, but nuclear localization of p16 in normal hepatocytes (denoted by **arrows**).
(TIF)

Figure S3 (**A**) Immunofluorescent detection of p16 in BL185 cells infected with either vector control or retrovirus encoding exogenous p16. (**B**) Leptomycin B (LMB) blocks the nuclear export of p16. Immunofluorescent staining for p16 in MM189 p16 cells demonstrates nuclear and cytoplasmic localization in untreated cells (left panel). Treatment with 5 nM LMB (Sigma) for 12 hours (middle panel) or 24 hours (right panel) results in nuclear accumulation.
(TIF)

Figure S4 (**A**) Migration activity of MM189 cells with ectopic p16 expression (MM189 p16) treated with Y27532 (Calbiochem), an inhibitor of RhoA. Data are from a representative experiment performed in duplicate. Bar, SEM. (**B**) Migration activity of MM189 cells with ectopic p16 expression (MM189 p16) treated with a Rac1 inhibitor (Calbiochem). Data are from a representative experiment performed in duplicate. Bar, SEM. *, *p*<0.05; **, *p*<0.01; ***, *p*<0.001.
(TIF)
