# Supplementary material for: p16 Stimulates CDC42-Dependent Migration of Hepatocellular Carcinoma Cells
Source: PLoS One. 2013 Jul 24;8(7):e69389. doi: 10.1371/journal.pone.0069389 (PMC3722281; doi:10.1371/journal.pone.0069389)
Supplement: File S1 — Supplementary methods. (DOC) [file pone.0069389.s001.doc]

**Supplementary Methods**

**shRNA clones**

mouse p16 (sh5, TRCN0000077817),

human p16 (sh1, TRCN0000039751; sh2, TRCN0000039752),

Cdc42 (sh1, TRCN0000331498; sh2, TRCN0000301724),

Rac1 (sh1, TRCN0000310888; sh2, TRCN0000301588),

RhoA (sh1, TRCN0000068200; sh2, TRCN0000068202),

Cdk4 (sh1, TRCN0000023174; sh2, TRCN0000023176),

Cdk6 (sh1, TRCN0000023151; sh2, TRCN0000023153),

pRb (sh1, TCRN0000295892; sh2, TRCN0000295841).

**Immunoblotting**

The following antibodies were used: anti-mouse p16 (sc-1207, Santa Cruz), anti-human p16 (sc-56330, Santa Cruz), anti-p19 (ab-80, Abcam), anti-p14 (sc-8340, Santa Cruz), anti-E-cadherin (610182, BD Transduction Lab), anti-α-catenin (610193, BD Transduction Lab), anti-β-catenin (610154, BD Transduction Lab), anti-N-cadherin (610920, BD Transduction Lab), anti-Vimentin (MS-129-P0, Thermo Scientific), anti-Twist (sc-15393, Santa Cruz), anti-Snail (3895, Cell Signaling), anti-Slug (Abgent, AP2053a), anti-Cdk4 (sc-23896, Santa Cruz), anti-Cdk6 (sc-53638, Santa Cruz), anti-RhoA (sc-418, Santa Cruz), anti-β-actin (sc-1615, Santa Cruz) and anti-α-tubulin (MS-581-P0, Fisher Scientific).
