# Supplementary material for: p16 Stimulates CDC42-Dependent Migration of Hepatocellular Carcinoma Cells
Source: PLoS One. 2013 Jul 24;8(7):e69389. doi: 10.1371/journal.pone.0069389 (PMC3722281; doi:10.1371/journal.pone.0069389)
Supplement: Table S1 — PCR primers used in vector construction. (DOCX) [file pone.0069389.s002.docx]

**Table S1. PCR primers used in vector construction**

| **Purpose** | **Name** | **Sequence** |
| --- | --- | --- |
| Mouse wild-type p16 | Forward primer with *BamH*I site | 5’AAGGATCCGCATGGGTCGCAGGTTCTTG |
|  | Reverse primer with *EcoR*I site | 5’GGGAATTCGCTTGAGCTGAAGCTATGCC |
| A12S mutant of p16 | Forward primer for the first PCR | 5’CTTTATCCAGCCCTCAC |
|  | Reverse primer for the first PCR | 5’CCTGGGCCGACGCCCTGG |
|  | Forward primer for the second PCR | 5’CCAGGGCGTCGGCCCAGG |
|  | Reverse primer for the second PCR | 5’ACCCTAACTGACACACATTCC |
| D66N mutant of p16 | Forward primer for the first PCR | 5’CTTTATCCAGCCCTCAC |
|  | Reverse primer for the first PCR | 5’GTAGTGGGGTTCTCGCAGTTC |
|  | Forward primer for the second PCR | 5’GAACTGCGAGAACCCCACTAC |
|  | Reverse primer for the second PCR | 5’ACCCTAACTGACACACATTCC |
| D76V mutant of p16 | Forward primer for the first PCR | 5’CTTTATCCAGCCCTCAC |
|  | Reverse primer for the first PCR | 5’CCGCGCTGCGACGTGCACCGG |
|  | Forward primer for the second PCR | 5’CCGGTGCACGTCGCAGCGCGG |
|  | Reverse primer for the second PCR | 5’ACCCTAACTGACACACATTCC |
| R79L mutant of p16 | Forward primer for the first PCR | 5’CTTTATCCAGCCCTCAC |
|  | Reverse primer for the first PCR | 5’GAAGCCTTCCAGCGCTGCGT |
|  | Forward primer for the second PCR | 5’ACGCAGCGCTGGAAGGCTTC |
|  | Reverse primer for the second PCR | 5’ACCCTAACTGACACACATTCC |
| E112K mutant of p16 | Forward primer for the first PCR | 5’CTTTATCCAGCCCTCAC |
|  | Reverse primer for the first PCR | 5’TGTCCCCGCTTTTGGGC |
|  | Forward primer for the second PCR | 5’GCCCAAAAGCGGGGACA |
|  | Reverse primer for the second PCR | 5’ACCCTAACTGACACACATTCC |
| p16 tagged with NES in N- and C-termini | Forward primer | 5’AAGGATCCATGCTGCCTCCTCTGGAGAGACTGACCCTGATGGAGTCCGCTGCA |
|  | Reverse primer | 5’GGGAATTCTCACAGGGTCAGTCTCTCCAGAGGAGGCAGGCTCTGCTCTTGGGA. |
| p16 tagged with NES in C-terminus | Forward primer | 5’AAGGATCCATCTGGAGCAGCATGGAGTC |
|  | Reverse primer | 5’GGGAATTCTCACAGGGTCAGTCTCTCCAGAGGAGGCAGGCTCTGCTCTTGGGA |
| p16 tagged with NLS in N- and C-termini | Forward primer | 5’AAGGATCCATGCCAAAGAAGAAGCGTAAGGTTATGGAGTCCGCTGCAGAC |
|  | Reverse primer | 5’GGGAATTCTTAAACCTTACGCTTCTTCTTTGGGCTCTGCTCTTGGGA |
| p16 tagged with NLS in C-terminus | Forward primer | 5’AAGGATCCATCTGGAGCAGCATGGAGTC |
|  | Reverse primer | 5’GGGAATTCTTAAACCTTACGCTTCTTCTTTGGGCTCTGCTCTTGGGA |
