# Supplementary figures and images for: p16 Stimulates CDC42-Dependent Migration of Hepatocellular Carcinoma Cells
Source: PLoS One. 2013 Jul 24;8(7):e69389. doi: 10.1371/journal.pone.0069389 (PMC3722281; doi:10.1371/journal.pone.0069389)

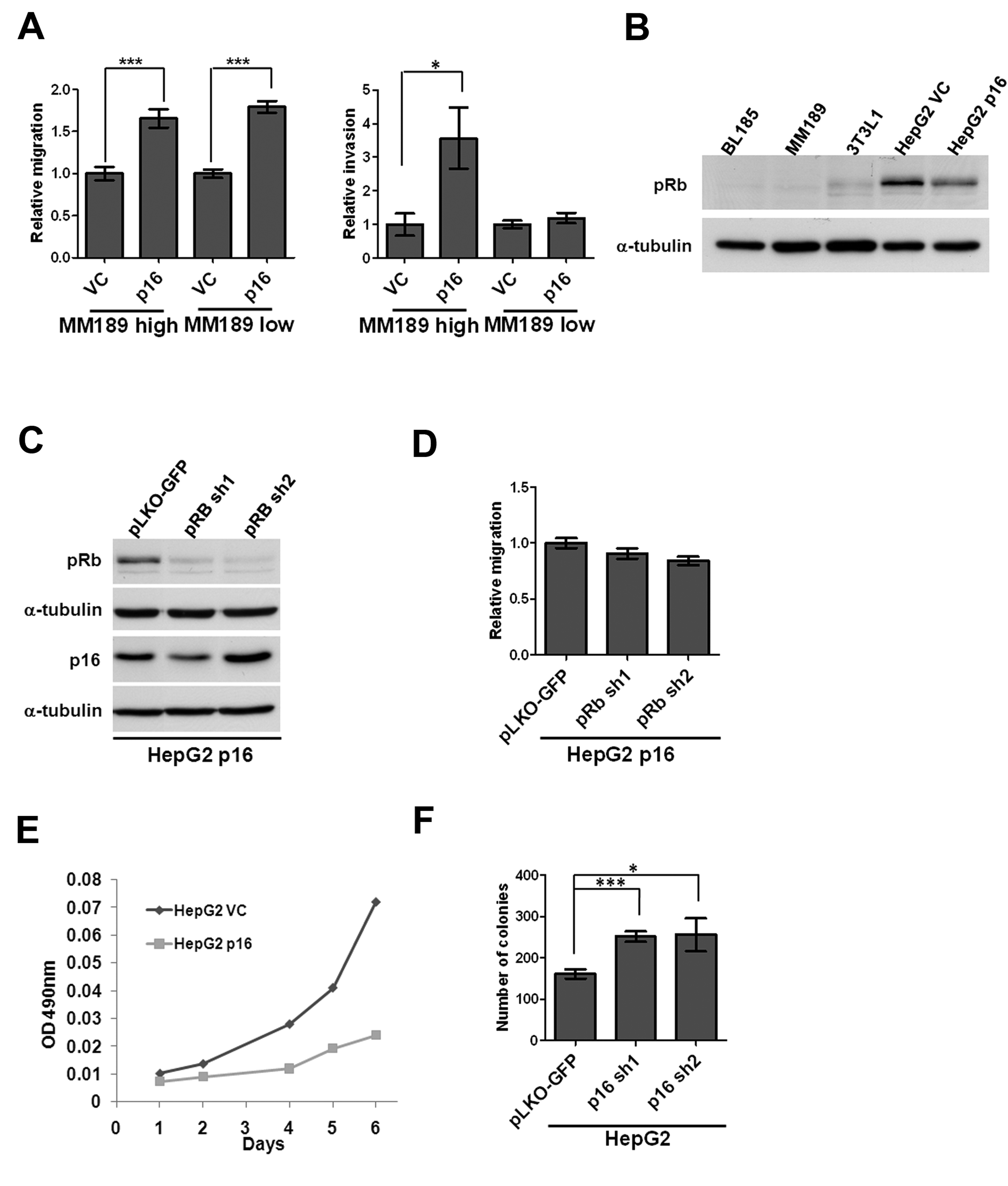

Supplement: Figure S1 — (A) Migration and invasion activity of low and high passage MM189 cells expressing p16, and their vector controls. Data are from representative experiments performed in duplicate. Bar, SEM. (B) Immunoblot detection of pRb expression (anti-Rb, sc-102, Santa Cruz) in MM189, BL322 and HepG2 cells. α-tubulin serves as a loading control. (C) Immunoblot detection of pRb knockdown in HepG2 cells with ectopic p16 expression (HepG2 p16). α-tubulin serves as a loading control. (D) Migration activity of HepG2 cells with ectopic p16 expression (HepG2 p16) following pRb knockdown, relative to cells expressing a non-silencing control (pLKO-GFP). Data are from a representative experiment performed in duplicate. Bar, SEM. (E) Representative cell proliferation assay for HepG2 with p16 ectopic expression (HepG2 p16) and vector control (HepG2 VC). Bar, SEM. (F) Representative soft agar assay for HepG2 cells with knockdown of p16, as well as the non-silencing control. Bar, SEM. *, p<0.05; **, p<0.01; ***, p<0.001. (TIF) [file pone.0069389.s003.tif]

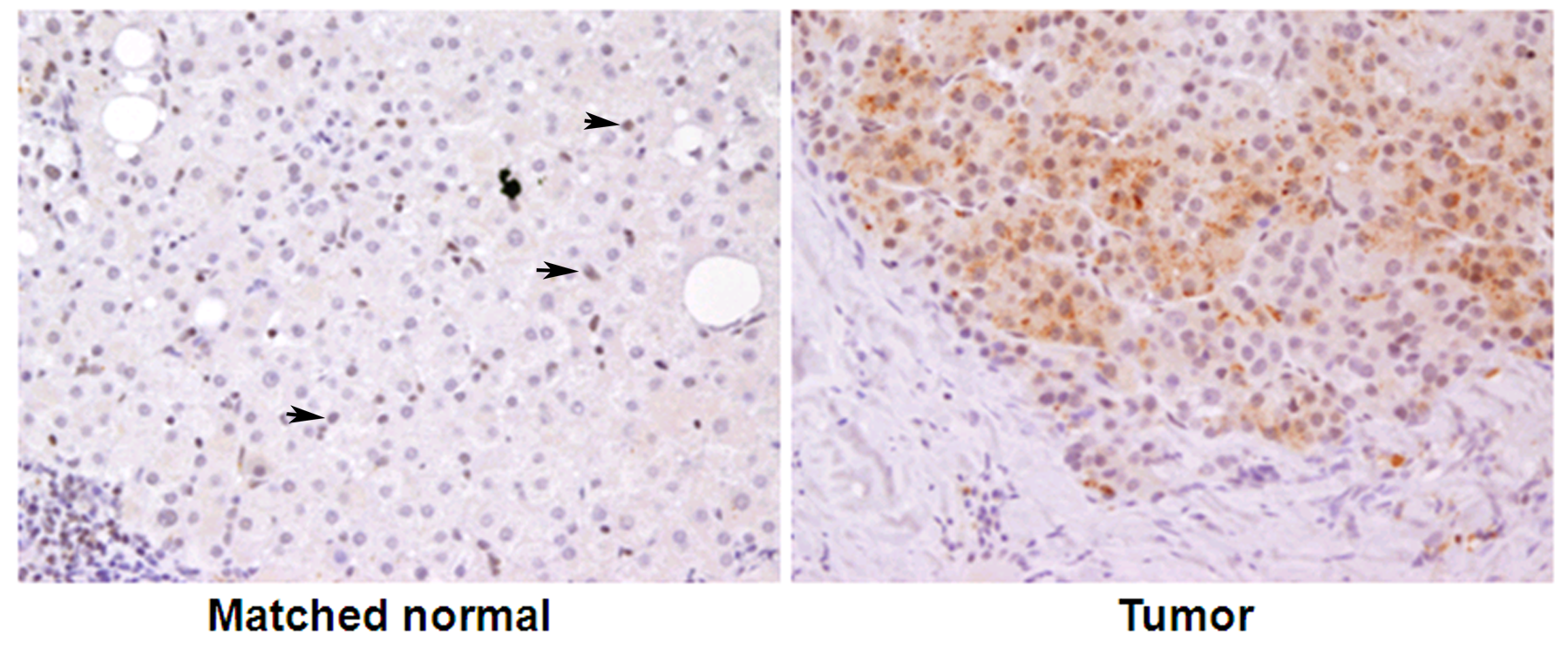

Supplement: Figure S2 — Immunohistochemical staining of paired HCC tissue and normal liver demonstrates cytoplasmic localization of p16 in the HCC specimen, but nuclear localization of p16 in normal hepatocytes (denoted by arrows). (TIF) [file pone.0069389.s004.tif]

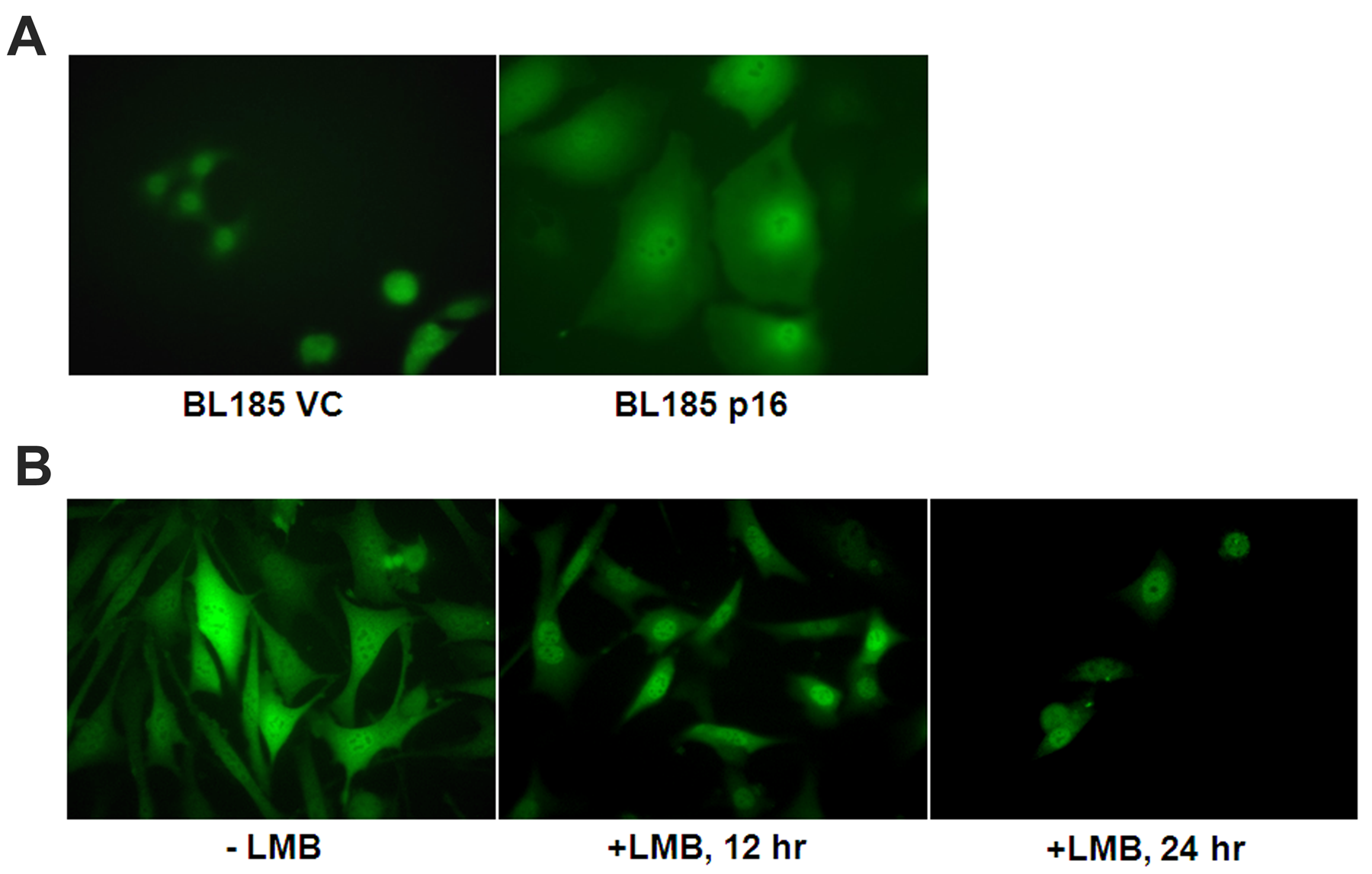

Supplement: Figure S3 — (A) Immunofluorescent detection of p16 in BL185 cells infected with either vector control or retrovirus encoding exogenous p16. (B) Leptomycin B (LMB) blocks the nuclear export of p16. Immunofluorescent staining for p16 in MM189 p16 cells demonstrates nuclear and cytoplasmic localization in untreated cells (left panel). Treatment with 5 nM LMB (Sigma) for 12 hours (middle panel) or 24 hours (right panel) results in nuclear accumulation. (TIF) [file pone.0069389.s005.tif]

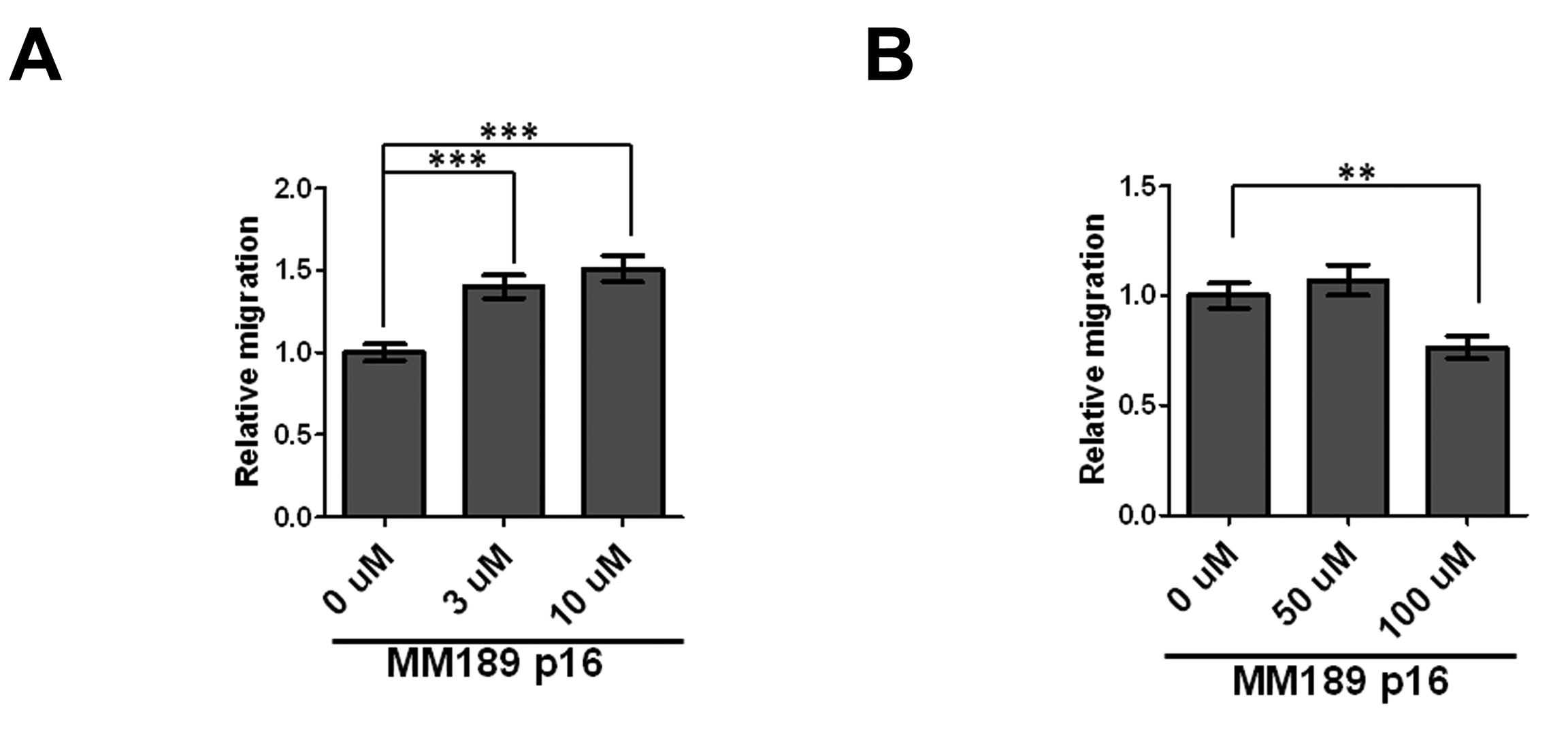

Supplement: Figure S4 — (A) Migration activity of MM189 cells with ectopic p16 expression (MM189 p16) treated with Y27532 (Calbiochem), an inhibitor of RhoA. Data are from a representative experiment performed in duplicate. Bar, SEM. (B) Migration activity of MM189 cells with ectopic p16 expression (MM189 p16) treated with a Rac1 inhibitor (Calbiochem). Data are from a representative experiment performed in duplicate. Bar, SEM. *, p<0.05; **, p<0.01; ***, p<0.001. (TIF) [file pone.0069389.s006.tif]
